# Prediction of Gestational Diabetes Mellitus and Pre-diabetes 5 Years Postpartum using 75 g Oral Glucose Tolerance Test at 14–16 Weeks’ Gestation

**DOI:** 10.1038/s41598-018-31614-z

**Published:** 2018-09-06

**Authors:** Tove Lekva, Kristin Godang, Annika E. Michelsen, Elisabeth Qvigstad, Kjersti Ringvoll Normann, Errol R. Norwitz, Pål Aukrust, Tore Henriksen, Jens Bollerslev, Marie Cecilie Paasche Roland, Thor Ueland

**Affiliations:** 1 Research Institute of Internal Medicine, Oslo University Hospital, Rikshospitalet, Oslo, Norway; 2 Section of Specialized Endocrinology, Department of Endocrinology, Oslo University Hospital, Rikshospitalet, Oslo, Norway; 3 Faculty of Medicine, University of Oslo, Oslo, Norway; 4 Department of Endocrinology, Morbid Obesity and Preventive Medicine, Oslo University Hospital, Aker, Oslo, Norway; 5 National Advisory Unit for Womens Health, Oslo University Hospital, Rikshospitalet, Oslo, Norway; 6 Mother Infant Research Institute, Tufts Medical Center, Boston, MA USA; 7 Department of Obstetrics & Gynecology, Tufts Medical Center and Tufts University School of Medicine, Boston, MA USA; 8 Section of Clinical Immunology and Infectious Diseases, Oslo University Hospital, Rikshospitalet, Oslo, Norway; 9 K.G. Jebsen Inflammatory Research Center, University of Oslo, Oslo, Norway; 10 K.G. Jebsen Thrombosis Research and Expertise Center, University of Tromsø, Tromsø, Norway; 11 Department of Obstetrics, Oslo University Hospital, Rikshospitalet, Oslo, Norway

## Abstract

Early detection and treatment of women at risk for gestational diabetes mellitus (GDM) could improve perinatal and long-term outcomes in GDM women and their offspring. We explored if a 75 g oral glucose tolerance test (OGTT) at 14–16 weeks of gestation could identify women who will (1) develop GDM or give birth to large-for-gestational-age (LGA) babies in 1031 pregnant women from the STORK study using different diagnostic criteria (WHO1999, IADPSG2010, WHO2013, NORWAY2017) and (2) develop pre-diabetes 5 years postpartum focusing on first trimester β-cell function in a separate study of 300 women from the STORK cohort. The sensitivity of the 14–16 week OGTT to identify women who would develop GDM or have LGA babies was low, and we could not identify alternative cut-offs to exclude women not at risk or identify women that could benefit from early intervention. First trimester β-cell function was a stronger determinant than third trimester β-cell function of predicting maternal pre-diabetes. In conclusion, in our normal low-risk population, the 75 g OGTT at 14–16 weeks is insufficient to identify candidates for early treatment of GDM or identify women not likely to develop GDM or have LGA babies. First trimester β-cell function may predict pre-diabetes 5 years postpartum.

## Introduction

Gestational diabetes mellitus (GDM), defined as glucose intolerance with onset or first recognition during pregnancy, affects 4–18% of pregnancies. It is caused by an imbalance between insulin resistance and secretion, and occurs when pancreatic β-cells fail to keep up with insulin production in the face of the increasing insulin resistance during pregnancy^1,2^. GDM is associated with obstetric complications with short- and long-term health consequences for the infant and increased long-term risk of cardio-metabolic disease in the mother. Criteria used to diagnose GDM vary with different glucose cut-offs and one or two-step approaches, making it difficult to compare studies and identify those pregnancies that are truly at risk. The Hyperglycemia and Adverse Pregnancy Outcome (HAPO) study^3^ outlined a uniform set of diagnostic criteria endorsed in 2010 by the International Association of the Diabetes and Pregnancy Study Group (IADPSG) and, in 2013, by the World Health Organization (WHO)^4^. These criteria have been accepted by most countries throughout the world, but have been criticized because of the large number of women who will have to undergo testing with implications on service provision, and the high number of women who will be diagnosed with GDM without have a real risk profile^5^. Countries such as the United Kingdom, Italy, and Norway perform risk factor-based screening, with only high-risk individuals receiving the 75 g oral glucose tolerance test (OGTT). In the United States, the American Diabetes Association (ADA) and the American Congress of Obstetricians and Gynecologists (ACOG) use a two-step approach with an initial universal screening test (50 g glucose load test) followed by a diagnostic test (100 g OGTT) at 24–28 weeks of gestation. Norway has recently modified their recommendations and are now using diagnostic criteria similar to that used in the US and Canada (Supplemental Table 1).

Treatment of GDM improves perinatal, obstetric and maternal outcomes^6^, but risk for long-term cardio-metabolic outcomes still remains high in the offspring of GDM mothers^7^, possibly due to prolonged exposure to maternal hyperglycemia. Using current criteria, testing for GDM may not be completed until 30 weeks, leaving a narrow window for intervention. Thus, early detection and treatment of women at risk for GDM, ideally at the time of enrollment in prenatal care, could improve perinatal and long-term outcomes in GDM women and their offspring^8,9^.

We hypothesized that a 75 g OGTT at 14–16 weeks could identify women who will subsequently develop GDM and this was examined in a prospective cohort study using four different diagnostic criteria for GDM: the WHO1999, IADPSG2010, WHO2013, and the new 2017 Norway criteria. We examined multiple potential cutoffs for early diagnosis of GDM and large for gestational age (LGA) babies and also assessed alternative markers of glucose metabolism derived from the OGTT as well as combinations with BMI and age. In a separate study, we examined the ability of first trimester β-cell function measurements to identify women with pre-diabetes at 5-years postpartum.

## Materials and Methods

We performed a secondary analysis of the STORK study, a prospective longitudinal cohort study in which 1031 low-risk women of Scandinavian heritage were followed throughout their pregnancy and gave birth at Oslo University Hospital Rikshospitalet between 2002 and 2008^10^. The exclusion criteria were multiple pregnancies, known pre-gestational diabetes and any severe chronic diseases (lung, cardiac, gastrointestinal or renal). Each pregnant woman had four study-related antenatal visits at weeks 14–16, 22–24, 30–32, and 36–38. A 75 g OGTT was performed on all women at 14–16 and 30–32 weeks of gestation. All women were invited to participate in a 5-year postpartum follow-up study of which 300 from the original study agreed^11^. Written informed consent was obtained from all study participants. All clinical investigations were conducted in accordance with the principles enshrined in the Declaration of Helsinki. The study was approved by the Regional Committee for Medical Research Ethics of Southern Norway in Oslo, Norway.

### Measurements of glucose and insulin from OGTT

All 75 g OGTTs were performed in the morning after an overnight fast and glucose levels measured as previously reported^11^. Briefly, venous blood was drawn in gel tubes, allowed to clot for 30 min, thereafter centrifuged for 10 min 3000 *g*, serum separated and stored at −80  °C. Glucose was measured in serum samples collected at antenatal visits at 14–16 and 30–32 weeks and frozen until analysis, using the hexokinase method at an accredited clinical chemistry laboratory at Oslo University Hospital (Cobas 6000 from Roche). For the 5-year follow-up visit, fasting glucose measurements were collected using an Accu-check Sensor glucometer (Roche Diagnostics), using venous EDTA blood analyzed on site, as previously reported^11^. Insulin levels were assayed in duplicate (RIA, DPC, Los Angeles, CA, USA) as previously reported^11,12^.

### Diagnosis of GDM and pre-diabetes

GDM was diagnosed using four separate criteria: (i) the WHO1999 criteria (2 h plasma glucose ≥7.8 mmol/L), (ii) the IADPSG2010 criteria (fasting plasma glucose (FPG) 5.1–6.9 mmol/L, 1 h plasma glucose ≥10.0 mmol/L or 2 h plasma glucose 8.5–11.0 mmol/L) and in addition FPG 5.1–6.9 mmol/L in early pregnancy, (iii) the WHO2013 criteria (fasting plasma glucose (FPG) 5.1–6.9 mmol/L, 1 h plasma glucose ≥10.0 mmol/L or 2 h plasma glucose 8.5–11.0 mmol/L, at any time in pregnancy) and (iv) Norway2017 criteria (FPG ≥ 5.3 mmol/L or 2 h plasma glucose ≥9.0 mmol/L)^13^ following a 75 g oral glucose load. Pre-diabetes was diagnosed at the 5-year follow-up visit using the following criteria: FPG 5.6–6.9 mmol/L or 2 h plasma glucose 7.8–11.0 mmol/L after 75 g OGTT^14^.

Insulin sensitivity was measured with the Matsuda index 10 000/√ of (fasting glucose (mmol/L) × fasting insulin (mU/L) × (mean glucose (mmol/L) × mean insulin (mU/L)) during 75 g OGTT. This index is a measure of whole body insulin sensitivity that has been validated against the euglycemic-hyperinsulinemic clamp^15^. β-cell function was assessed with the insulin secretion-sensitivity index (ISSI-2) (area under the curve insulin(mU/L)_0–120_/glucose(mmol/L)_0–120_ × Matsuda), validated against the disposition index from the intravenous glucose tolerance test^16^. HOMA-IR was calculated as fasting insulin (mU/L) × fasting glucose (mmol/L)/22.5, as described by Matthews *et al*.^17^. Insulin secretion was assessed by the insulinogenic index (IGI: (Ins30–Ins0)/(Glu30–Glu0)).

### Statistical analysis

Statistical analyses were conducted using SPSS for Windows, version 21.0. Data are expressed as mean (SD) when normally distributed and median (25^th^, 75^th^ percentile) when skewed. Comparison between women with GDM and non-GDM were performed using *t*-test or Mann–Whitney’s *U* depending on distribution, and *χ*^2^ test for categorical variables. Accuracy was represented using the terms sensitivity, specificity, positive predictive value (PPV), and negative predictive value (NPV) calculated as follows: Sensitivity = True positive/(True positive + False negative), Specificity = True negative/(False positive + True negative), Positive predictive value (PPV) = True positive/(True positive + False positive), Negative predictive value (NPV) = True negative/(False negative + True negative). The association between glucose levels and accuracy terms was assessed using third order polynomial regression. Receiver operator characteristics (ROC) analysis was used to determine the optimum cut-off values for some markers. p < 0.05 was considered statistically significant.

## Results

**Figure 1:**
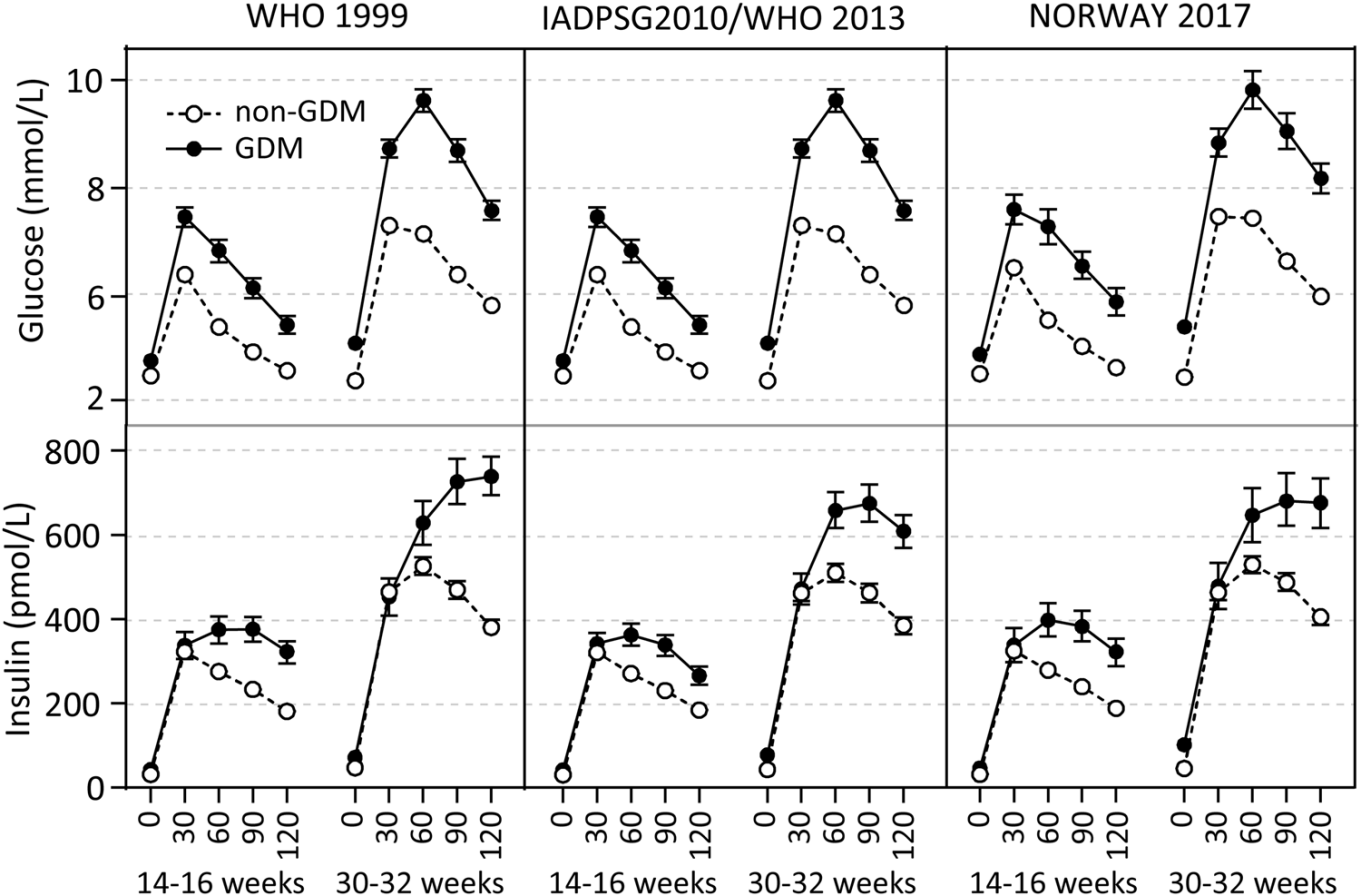
OGTT data. Glucose and insulin levels during OGTT at weeks 14–16 and 30–32 in pregnancy between GDM (black circles) and non-GDM (white circles). Data presented as mean (95% CI).

Of the 1031 women in the study with representative OGTT results at 30–32 weeks, we identified 132 (13.4%) women who developed GDM based on the WHO1999 criteria, 241 (24.5%) based on the IADPSG2010, 244 (24.8%) based on the WHO2013 criteria, and 91 (9.2%) based on Norway criteria compared to 853, 744, 741, and 896 control women without GDM, respectively. Their clinical characteristics are provided in Table 1. In general, women who developed GDM were older, had higher BMI, higher blood pressure (BP), and higher proportion of LGA babies. As expected, we found higher insulin resistance and area under the curve (AUC) for glucose and insulin, and lower insulin sensitivity and β-cell function, as assessed by insulin secretion-sensitivity index, in women diagnosed with GDM at weeks 14–16 and 30–32 (Table 1). The glucose and insulin measurements from the OGTT at weeks 14–16 and 30–32 are presented in Fig. 1. The correlation of fasting glucose and the four post-load glucose measurements during OGTT at week 14–16 and corresponding measurement at week 30–32 were, r = 0.54, r = 0.48, r = 0.54, r = 0.58 and r = 0.50, respectively (p < 0.001).

**Table 1:** Characteristics of mothers with GDM versus non-GDM.

	WHO 1999 (2 h glucose ≥7.8)		IADPSG 2010 $ (Fasting, 1 h, or 2 h glucose ≥5.1, ≥10, ≥8.5)		WHO 2013# (Fasting, 1 h, or 2 h glucose ≥5.1, ≥10, ≥8.5)		Norway 2017 (Fasting or 2 h glucose, ≥5.3, ≥9.0)	
	Non-GDM	GDM	Non-GDM	GDM	Non-GDM	GDM	Non-GDM	GDM
N=	853	132	744	241	741	244	896	91
Age (years)	31.2 (3.8)	32.0 (4.3)*	31.0 (3.7)	32.0 (4.3)*	31.0 (3.7)	32.0 (4.3)*	31.0 (3.8)	31.8 (4.5)
Height (cm)	169 (6)	168 (5)*	169 (6)	168 (6)	169 (6)	168 (6)	169 (6)	169 (6)
Fam history diabetes n (%)	87 (10.2)	12 (9.1)	73 (9.8)	25 (10.4)	73 (9.9)	25 (10.2)	93 (10.4)	6 (6.6)
Primipara n (%)	456 (53.5)	67 (50.8)	415 (56.2)	111 (46.6) *	413 (56.2)	112 (46.5)*	485 (54.6)	40 (44.4)
Preeclampsia n (%)	28 (3.3)	7 (5.3)	25 (3.4)	10 (4.1)	25 (3.4)	9 (3.7)	32 (3.6)	3 (3.3)
LGA/SGA (%)	107 (12.5)/62 (7.3)	28 (21.2)*/8 (6.1)	82 (11.1)/62 (8.4)	53 (22.0)*/9 (3.7)	81 (11.0)/62 (8.4)	54 (22.1)*/9 (3.7)	112 (12.5)/66 (7.4)	23 (25.3)*/5 (5.5)
Currently smoking n (%)	17 (2.0)	6 (4.5)	14 (2.4)	9 (5.1)	14 (2.4)	10 (5.5)*	18 (2.6)	5 (7.8)*
Previous smoker n (%)	183 (21.5)	37 (28.0)	156 (21.4)	63 (27.2)	156 (21.5)	63 (26.9)	193 (22.0)	27 (31.4)*
BMI (kg/m^2^)^a^	23.8 (21.7, 26.0)	27.9 (25.0, 31.1)*	23.5 (21.5, 25.7)	25.5 (23.1, 28.5)*	23.5 (21.5, 25.7)	25.4 (23.1, 28.4)*	23.7 (21.7, 26.0)	26.4 (23.8, 30.5)*
BMI (kg/m^2^)^b^	26.5 (24.3, 29.1)	27.9 (25.0, 31.1)*	26.2 (24.0, 28.6)	28.4 (26.2, 31.1)*	26.2 (24.0, 28.6)	28.4 (26.2, 31.1)*	26.5 (24.2, 28.9)	29.0 (26.4, 32.4)*
Systolic BP (mmHg)^a^	110 (100, 120)	110 (105, 120)*	110 (100, 120)	110 (105, 120)*	110 (100, 120)	110 (105, 120)*	110 (100, 120)	115 (110, 120)*
Systolic BP (mmHg)^b^	110 (105, 120)	110 (110, 120)	110 (105, 120)	115 (110, 120)*	110 (105, 120)	115 (110, 120)*	110 (105, 120)	115 (110, 120)*
Diastolic BP (mmHg)^a^	65 (60, 70)	70 (60, 70)*	65 (60, 70)	70 (60, 70)*	65 (60, 70)	70 (60, 70)*	69 (60, 70)	70 (60, 70)*
Diastolic BP (mmHg)^b^	70 (60, 75)	70 (60, 75)	70 (60, 75)	70 (60, 75)*	70 (60, 75)	70 (60, 75)*	70 (60, 75)	70 (65, 75)*
Insulin sensitivity^a^	210 (149, 297)	135 (89, 203)*	217 (155, 307)	141 (93, 209)*	217 (156, 308)	140 (92, 210)*	206 (148, 293)	114 (81, 197)*
Insulin sensitivity^b^	114 (80, 169)	70 (49, 96)*	120 (84, 175)	71 (51, 101)*	120 (84, 174)	72 (51, 101)*	113 (79, 165)	59 (43, 79)*
Insulin resistance^a^	0.75 (0.50, 1.12)	1.04 (0.73, 1.71)*	0.71 (0.46, 1.05)	1.08 (0.77, 1.78)*	0.71 (0.46, 1.05)	1.07 (0.77, 1.78) *	0.76 (0.5, 1.12)	1.21 (0.84, 1.84) *
Insulin resistance^b^	1.12 (0.73, 1.73)	1.80 (1.19, 2.54)*	1.05 (0.69, 1.58)	1.84 (1.24, 2.61)*	1.05 (0.69, 1.58)	1.84 (1.24, 2.62)*	1.12 (0.74, 1.68)	2.30 (1.78, 3.62)*
β-cell function^a^	1140 (916, 1497)	805 (608, 1055)*	1190 (953, 1543)	821 (653, 1062)*	1193 (958, 1543)	817 (652, 1061)*	1133 (914, 1487)	737 (617, 908)*
β-cell function^b^	927 (723, 1187)	574 (456, 687)*	980 (780, 1247)	606 (485, 719)*	980 (781, 1247)	606 (484, 719)*	917 (719, 1165)	460 (402, 550)*
AUC glucose^a^	21.1 (18.7, 24.1)	26.2 (22.7, 30.7)*	20.6 (18.4, 23.7)	24.5 (22.1, 27.8)*	20.6 (18.5, 23.7)	24.6 (22.1, 28.0)*	21.3 (18.8, 24.2)	25.9 (22.7, 29.6)*
AUC glucose^b^	26.5 (23.4, 29.2)	34.1 (31.7, 36.3)*	26.0 (23.1, 28.6)	32.9 (29.2, 35.4)*	26.0 (23.1, 28.6)	32.8 (29.2, 35.3)*	26.8 (23.7, 29.7)	34.6 (30.5, 37.8)*
AUC insulin^a^	810 (605, 1156)	1099 (822, 1624)*	795 (596, 1125)	1006 (768, 1520)*	792 (597, 1120)	1006 (767, 1522)*	822 (615, 1163)	1164 (768, 1661)*
AUC insulin^b^	1528 (1089, 2075)	2046 (1400, 2741)*	1478 (1085, 2020)	1927 (1338, 2684)*	1478 (1087, 2017)	1912 (1316, 2684)*	1538 (1105, 2126)	1912 (1371, 2766)*

Data given as mean (SD) when normal distributed and median (25^th^, 75^th^) when skewed distributed. *p < 0.05 ^a^weeks 14–16, ^b^weeks 30–32. ^#^At any time in pregnancy. ^$^Fasting glucose 5.1–6.9 mmol/L in early pregnancy is also used to diagnose GDM.

### Early diagnosis of GDM when applying diagnostic criteria at 14–16 weeks

Based on the results of the 75 g OGTT at 14–16 weeks, we identified 20 (2.0%), 118 (12.5%), 119 (12.6%) and 46 (4.7%) women with GDM, using the WHO1999, IADPSG2010, WHO2013, and 2017 Norwegian criteria, respectively. The FPG in early pregnancy is included in the IADPSG2010 criteria and the OGTT used to diagnose GDM could be measured at any time using the WHO2013 criteria, so these criteria are using the 14–16 weeks OGTT as well. The PPV of this early OGTT for identifying women diagnosed with GDM was 95% using the WHO1999 criteria, 87.3% using the IADPSG2010, 89.9% using the WHO2013 criteria, and 47.8% using the 2017 Norwegian criteria. The NPV was 88.2%, 84.3%, 84.4% and 92.7%, respectively. The sensitivity and specificity were 14.8% and 99.9% for the WHO1999 criteria, 44.2% and 97.9% for the IADPSG2010, 45.3% and 98.3% for the WHO2013 criteria, and 25.0% and 97.2% for the 2017 Norwegian criteria (Supplemental Table 2).

### Examination of multiple cutoffs in the OGTT for early diagnosis of GDM

**Figure 2:**
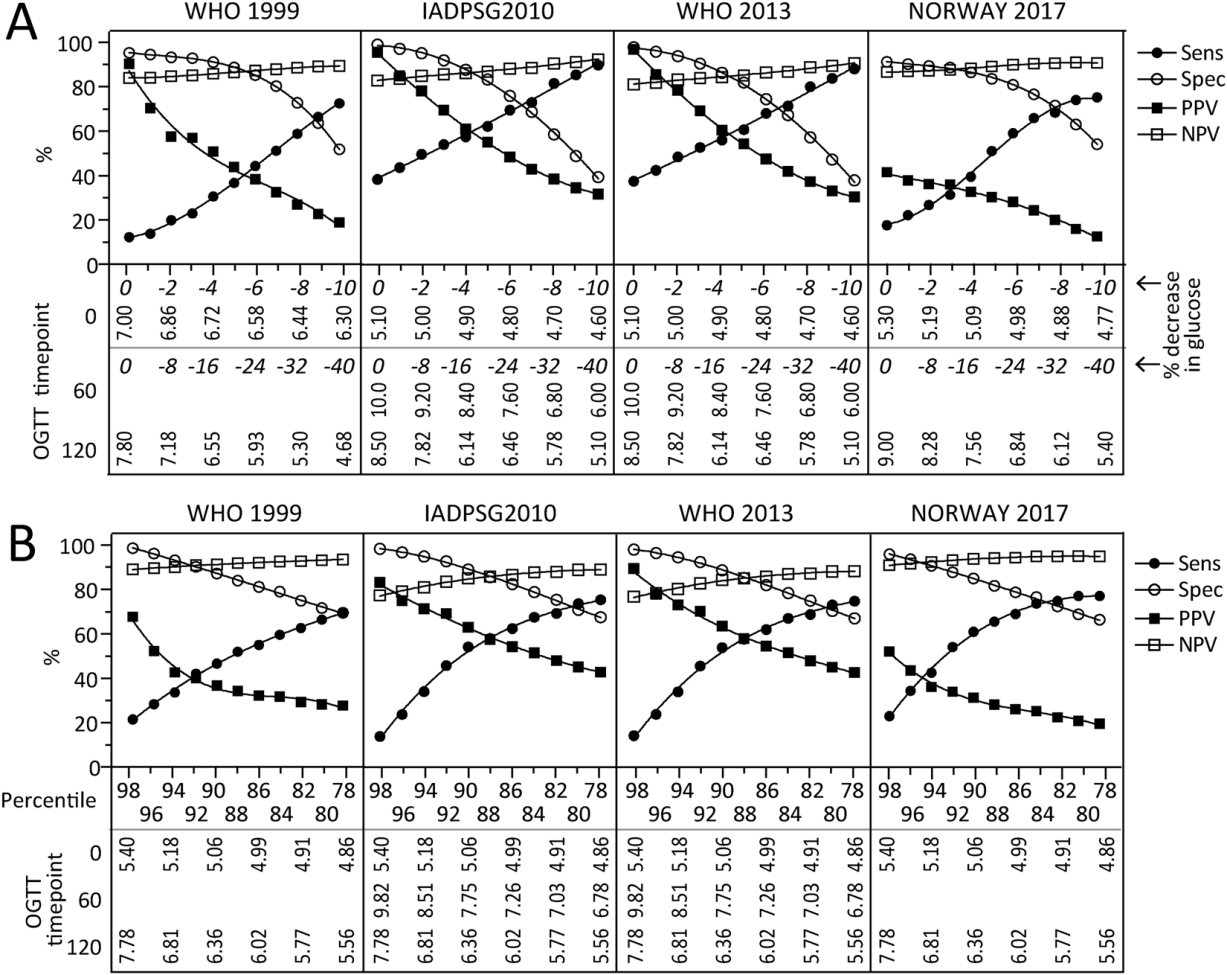
Test characteristics of different cut-offs of glucose from the 75 g OGTT at 14–16 weeks. Sensitivity, specificity, PPV and NPV across the different GDM diagnostic criteria using (**A**) 1% (fasting) and 4% (60 and 120 minutes) decreases from standard 30–32 week OGTT cut-offs, and (**B**) percentiles of glucose derived from the total population at 14–16 weeks in pregnancy. In (**A**) the % decrease in glucose is shown in italic. Vertical text shows the actual glucose values at the different time-points.

Compared with measurements at 14–16 weeks, measurements at 30–32 weeks in women with GDM (diagnosed at 30–32 weeks) were an average of 8.2% higher at baseline (i.e., fasting levels), 36.0% higher at 60 minutes, and 35.0% higher at 120 minutes during OGTT, regardless of the diagnostic criteria used. To evaluate the effect of lower diagnostic cut-offs and adjust for gestational age difference, we assessed the ability of the 75 g OGTT at 14–16 weeks to predict GDM when incrementally down adjusting the cut-offs by 1% (fasting) and 4% (60 and 120 minutes) from the original diagnostic criteria. As shown in Fig. 2A, the juncture between sensitivity and specificity (i.e. when they cross) for diagnosing GDM at 14–16 weeks was around a 7–9% and 28–32% decrease in fasting and 60/120 minutes glucose, respectively, giving a sensitivity and specificity around 70%. Approximately 80% sensitivity could be achieved when lowering fasting glucose by 8% and 60/120 minutes glucose by 32% for IADPSG2010 and WHO2013 while a slightly larger reduction (fasting glucose 10% and 60/120 minute glucose 40%) was needed for WHO1999 and Norway2017. However, at this extensive decrease in the glucose thresholds, around 40–50% of the population would be classified as at risk. Similar results were obtained when plotting test characteristics against percentiles of glucose derived from OGTT (from the total population) at 14–16 weeks (Fig. 2B).

### Combination of age and BMI with OGTT at 14–16 weeks in early diagnosis of GDM

To assess the impact of combining additional criteria together with glucose data from the OGTT at 14–16 weeks in predicting GDM at 30–32 weeks we evaluated combinations with BMI using a cut-off of 30 and with age using a cut-off of 35. For BMI, 87 women (9%) had a BMI over 30 and for age, 19.4% were 35 or older. The diagnostic accuracy of these measures alone varied somewhat depending on diagnostic criteria and is given in Supplemental Tables 3 and 4. Supplemental Fig. 1 shows how combining these measures with data from the OGTT as described above. Across the different diagnostic criteria, addition of BMI and/or age increased sensitivity modestly when using high cut-offs from the OGTT but there was little overall gain when evaluating lower glucose cut-offs. Specificity decreased accordingly with inclusion of these additional data and the PPV was poor in all models.

### Evaluation of alternative markers derived from the OGTT for early prediction of GDM

**Figure 3:**
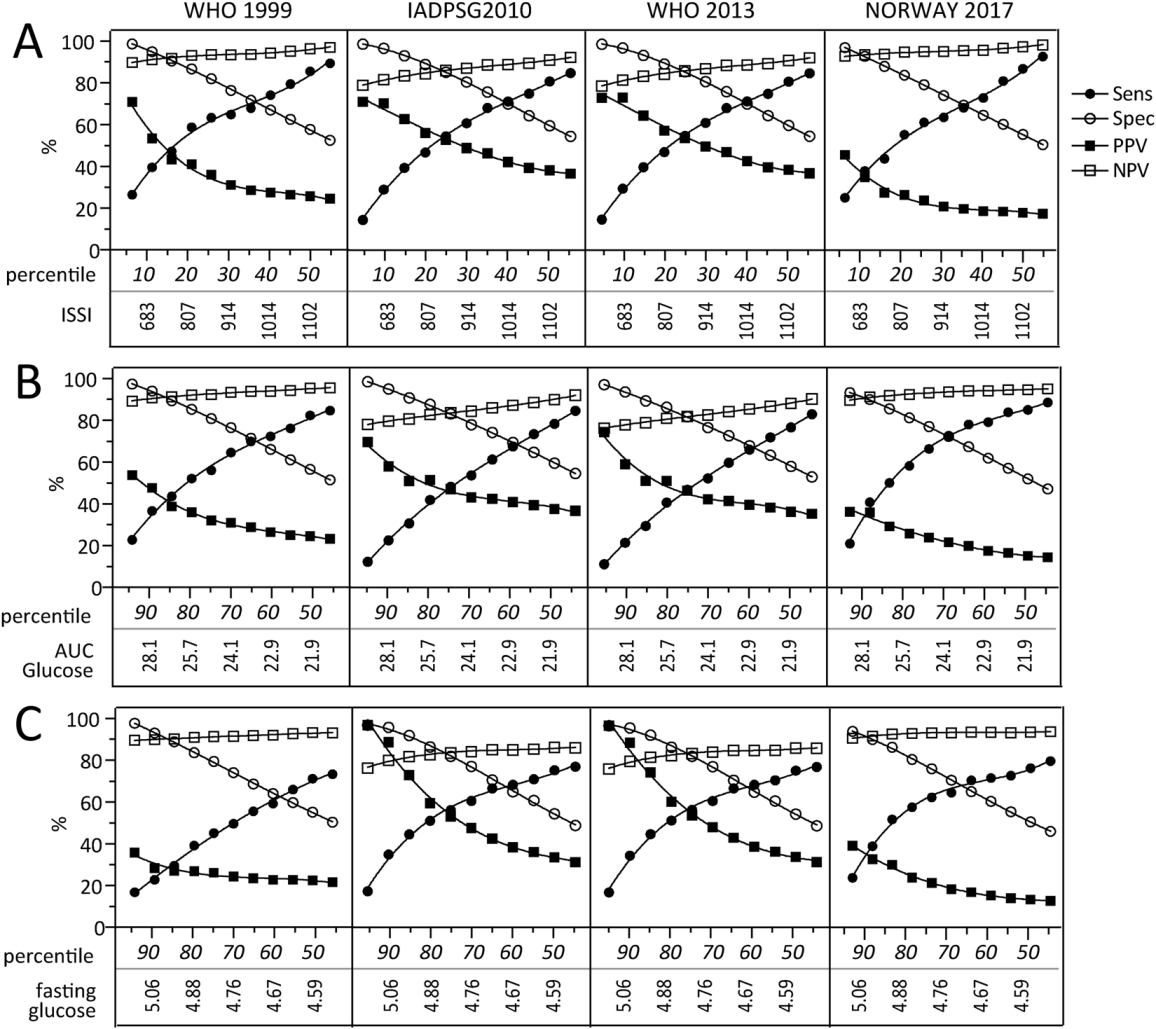
Test characteristics of different cut-offs based on percentiles obtained from the total population for β-cell function, AUC glucose, and fasting glucose from the 75 g OGTT at 14–16 weeks. Sensitivity, specificity, PPV and NPV across the different GDM diagnostic criteria using 5% changes in percentiles for (**A**) β-cell function, (**B**) AUC glucose and (**C**) fasting glucose at 14–16 weeks in pregnancy. The percentiles are shown in italic and vertical text shows the cut-off levels at the different time-points.

We next assessed the discriminatory properties of single measurements and AUC of glucose and insulin from the 14–16 week OGTT to predict GDM at 30–32 weeks using ROC analysis. As shown in Supplemental Fig. 2, the accuracy varied for the different glucose measurements, but was in general fair for most measures (i.e., AUC 0.7–0.8), with poorer performance for insulin. The best performance was in general achieved for β-cell function and AUC calculated for glucose during OGTT, although fasting glucose at 14–16 weeks had a high AUC for IADPSG2010 and WHO2013 since it is included in the diagnostic criteria. Figure 3 shows the different accuracy measures plotted against percentiles of β-cell function (panel A), AUC glucose (panel B) and fasting glucose (panel C) determined in the whole population. The cut-off to achieve a sensitivity of >80% using β-cell function or AUC glucose to predict GDM at 30–32 weeks is around the 50^th^ percentile, although it was higher for AUC glucose using the 2017 Norwegian criteria. At this cut-off, specificity is around 60% and the PPV is between 20–40%. Similarly, the sensitivity achieved using the 50^th^ percentile for fasting glucose (Fig. 3C) was 70–75% for the different criteria, with specificity around 50% and PPV between 20–30%. As fasting glucose at 14–16 weeks is included in the criteria for IADPSG2010 and WHO2013, a better PPV was achieved with these criteria.

### Predicting large for gestational age (LGA) infants using early OGTT data

**Figure 4:**
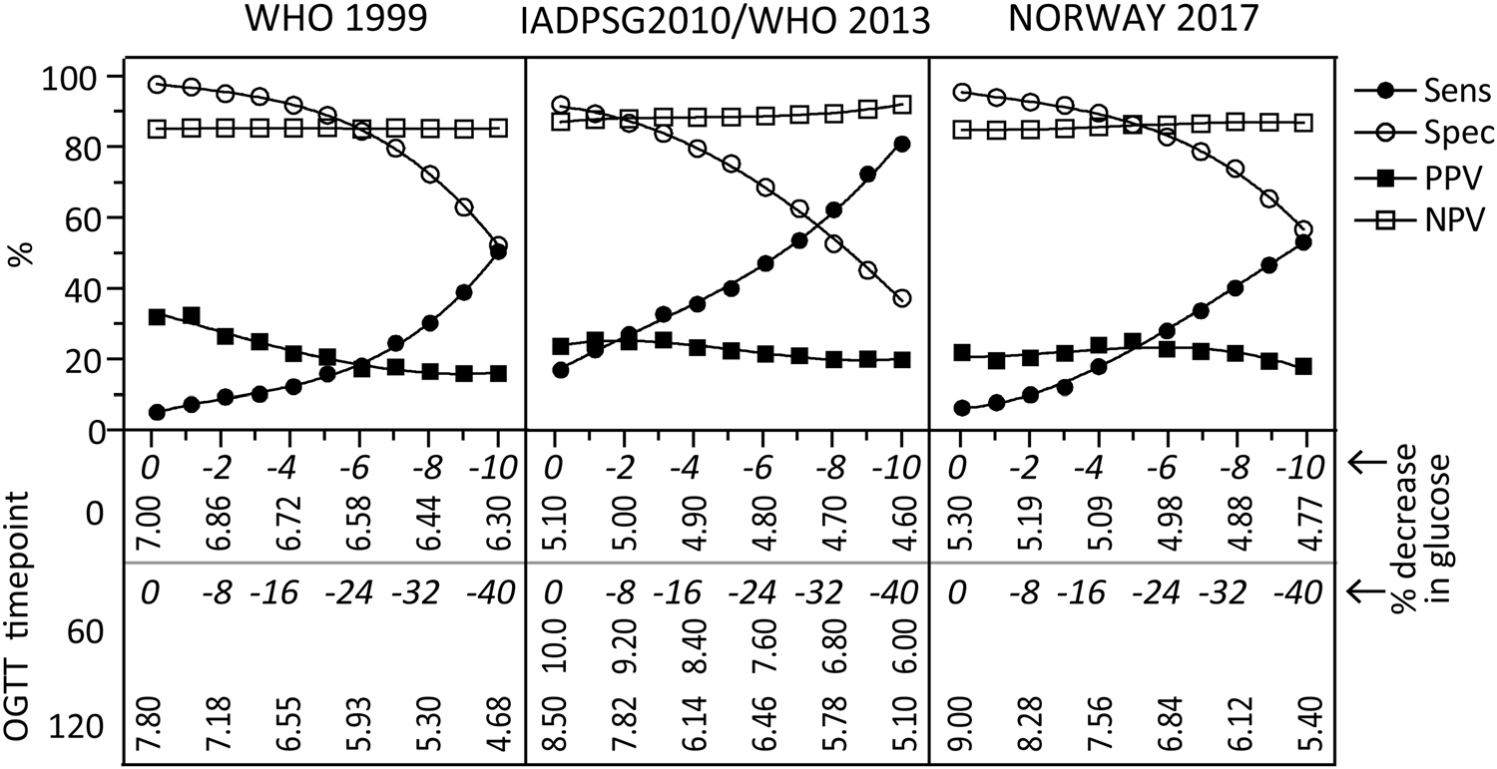
Test characteristics of different cut-offs of glucose from the 75 g OGTT at 14–16 weeks for identifying LGA children. Sensitivity, specificity, PPV and NPV across the different GDM diagnostic criteria using 1% (fasting) and 4% (60 and 120 minutes) decreases from standard 30–32 week OGTT cut-offs. The % decrease in glucose is shown in italic. Vertical text shows the actual glucose values at the different time-points.

142 (13.8%) of the women gave birth to a LGA infant. We found 35%, 23.7% and 18.6% of the GDM diagnosed at 14–16 weeks (diagnostic criteria using at standard 24–28 weeks) to deliver LGA babies diagnosed with the WHO1999, IADPSG2010/WHO2013 and Norway2017 criteria. This gave an OR of 3.24 (95% CI, [1.27–8.29]) p = 0.014 for WHO1999, OR 1.98 (95%CI, [1.23–3.20])) p = 0.005 for IADPSG2010/WHO2013 and 1.36 (95%CI, [0.61–2.99]) p = 0.452 for the Norway2017 criteria. The diagnostic accuracy for detecting LGA babies by applying the different diagnostic criteria at 14–16 weeks is given in Supplemental Table 5 and shows a sensitivity of 5–6% for WHO1999 and Norway2017 criteria, and a sensitivity of 20.1% using IADPSG2010/WHO2013 criteria. Figure 4 shows the diagnostic accuracy when lowering the thresholds of these criteria. For WHO1999 and Norway2017, sensitivity remained quite poor, while 80% sensitivity could be achieved for IADPSG2010/WHO2013 when lowering fasting glucose by 10% and 60/120 minutes glucose by 40% but at this extensive decrease in glucose more than half of the population would have a positive test.

### Second study: Predicting pre-diabetes using early estimates of β-cell function

We have previously identified β-cell dysfunction in the third trimester as a strong predictor of pre-diabetes at 5-years postpartum^11^. In this study, we examined whether early estimates of β-cell function could predict pre-diabetes 5 years after the index pregnancy, using a separate dataset of 300 women who completed the 5-year follow-up visit, of which 20 had pre-diabetes. Evaluated as a continuous variable, log β-cell function at 14–16 weeks could identify women at risk of developing pre-diabetes (OR 0.34 [95% CI, 0.20–0.58], p < 0.001 per SD change). Similar values were obtained for β-cell function at 30–32 weeks (OR 0.42 [0.26–0.67], p < 0.001 per SD change). A comparison of both variables revealed that β-cell function at 14–16 weeks was a stronger predictor (14–16 weeks: OR 0.41 [0.22–0.76], p = 0.005 per SD change vs 30–32 weeks: OR 0.76 [0.42, 1.37], p = 0.36 per SD change). No colinearity issues were identified when including β-cell function at 14–16 and 30–32 weeks in the same model (tolerance 0.60, VIF 1.68).

We next evaluated if the combination of early β-cell dysfunction and later GDM was associated with higher risk for future pre-diabetes. The association with pre-diabetes was dependent on diagnostic criteria. No association between GDM and pre-diabetes using the WHO1999 criteria was detected. For IADPSG2010 and WHO2013, the combination with GDM did not enhance prediction of pre-diabetes (i.e. lower total Wald score). However, when the interaction between β-cell function and GDM was included in the model, the association improved. For Norway2017, β-cell function and GDM were associated with future pre-diabetes with a combined Wald score similar to β-cell function alone (Supplemental Table 6).

## Discussion

Early detection of GDM could enhance the opportunity for interventions to improve maternal and neonatal outcomes. We evaluated the ability of measurements obtained from 75 g OGTT performed at 14–16 weeks in normal low-risk pregnancies to predict a diagnosis of GDM at 30–32 weeks. While information from the OGTT had a potential to identify women who will develop GDM, applying the original GDM criteria early in pregnancy had a very poor sensitivity and while specificity was high, the majority of GDM women would be missed, limiting its usefulness both as a screening tool to identify women for later follow-up and for selecting women who would benefit from early intervention, as a substantial number of non-GDM women would need to be treated. Lowering the diagnostic cut-offs, combining with other diagnostic criteria such as BMI and age, or assessing alternative measures of glucose metabolism did not enhance diagnostic accuracy in a clinically useful way. The diagnostic accuracy for identifying LGA children was poorer than for GDM. Our data obtained from a prospectively sampled cohort do not support early testing for GDM in normal low-risk pregnancies using OGTT. In the second study, early estimates of β-cell function predicted pre-diabetes at 5 years postpartum.

Previous studies investigating fasting glucose in early pregnancy using ROC analysis to predict future GDM^18,19^ found AUC between 0.67 and 0.72, with a high sensitivity (80%) but a poor specificity (high false positive rate of 53–57%). These studies concluded that fasting glucose in early pregnancy is an inefficient screening test for GDM. Our ROC analysis showed an AUC of 0.64–0.76 with a sensitivity of 65–70% and a false positive rate of 24–47% for fasting glucose, depending on the diagnostic criteria used, which is consistent with these prior publications. Additional studies have looked at glucose measurements at other time-points during the OGTT to see whether these might yield better diagnostic performance. Phaloprakarn *et al*.^20^ found that the 1 h glucose level with a cut-off value of 8.6 mmol/L gave a good diagnostic accuracy, but with low specificity (64.3%) and a poor PPV (38.9%). They concluded that this too was an inappropriate test to identify women who were likely to develop GDM later in pregnancy. In our study, applying the cut-offs of different criteria for GDM diagnosis early in pregnancy revealed a high specificity, although many cases of GDM would be missed due to the low sensitivity. Increasing sensitivity by lowering the cut-offs of glucose measurements at baseline (fasting), 60 and 120 minutes, revealed that a modest sensitivity of around 40–70% could be achieved. The association between other measures derived from the OGTT at 14–16 weeks and a later GDM diagnosis varied somewhat, but in general β-cell function and AUC glucose performed best but gave similar diagnostic performance as using cut-off from the OGTT. Previously, the insulinogenic index has been associated with postpartum glucose intolerance^21^ and in the present study, early assessment of insulinogenic index was associated with a GDM diagnosis later in pregnancy but did not perform as well as other indices calculated from the OGTT. Thus, to obtain this modest sensitivity, 40–50% of the population would be identified as at risk and require additional testing later in pregnancy. Such a strategy—namely, early OGTT screening to exclude women at very low risk, followed by rescreening in a proportion of women—would markedly increase the total number and burden of OGTTs performed and an unacceptable proportion of GDM women would be missed. Furthermore, to obtain a high specificity for identifying women who could benefit from early intervention, a substantial number of non-GDM women would also be treated.

Previous studies evaluating the ability for early OGTT data to predict GDM have focused on one diagnostic criteria, often in high-risk patients and have not thoroughly evaluated the different diagnostic accuracy measures of glucose data. We extend these previous studies by evaluating different diagnostic criteria and cut-offs for glucose during OGTT and integrated measure of glucose metabolism in a large cohort of prospectively sampled apparently healthy women. Thus, data obtained from OGTT early in pregnancy seem inadequate to identify candidates both for screening purposes and early treatment of GDM. Combination of early OGTT data and demographics such as BMI and age increased sensitivity and showed some merit for all criteria except the WHO1999 criteria and combinations of early OGTT data in combination with other biomarkers should be pursued in future studies. Although fasting glucose performed less well than measures calculated from the OGTT, it did not perform that much poorer when evaluated as a screening test to exclude the likelihood of developing GDM. Due to the simplicity of obtaining a fasting glucose sample, we cannot exclude that some benefit could be achieved with a strategy utilizing early measurement of fasting glucose or use of other clinical data in combination with OGTT data followed by an OGTT later in pregnancy.

A third trimester OGTT remains the “gold standard” for the diagnosis of GDM. The utility of a screening test for GDM is highly debated and many questions remain unanswered, such as: Should we screen? Should we use a one or two-step approach? What is the most cost effective approach? What biochemical screening test is best?^22^. The different international diagnostic criteria used and the ongoing debate on which should be the “gold standard” complicates comparison of studies and research for improving care for women with GDM. In the present study we employed four different criteria for GDM diagnosis, and regardless of the criteria used, our data suggest that an early OGTT has limited clinical benefit. The IADPSG2010/WHO2013 was adopted by many after the HAPO study established a relationship between maternal hyperglycemia and adverse outcomes, in particular LGA^3^. Iwama *et al*. did not identify a strong association between data obtained from an early OGTT and LGA^23^. Our study shows that the WHO1999 and in particular the IADPSG2010/WHO2013 criteria are associated with LGA also when applied early in pregnancy. However, similar to establishing an early GDM diagnosis, the diagnostic accuracy is quite poor and clinical usefulness limited.

A diagnosis of GDM is associated with increased risk of developing type 2 diabetes later in life^24^. We and others^11,25^ have identified poor β-cell function in the second and/or third trimester as a predictor of pre-diabetes during long-term follow-up. A recent meta-analysis identified an association between early diagnosis of GDM and risk of type 2 diabetes^26^, suggesting that the link between GDM and risk of type 2 diabetes may manifest early in pregnancy. The second part of the present study supports this assertion and shows that declining β-cell function already in the first trimester is associated with increased risk for pre-diabetes at 5-years postpartum, and is an even stronger predictive test than β-cell function estimated in the third trimester. Furthermore, the combination of a later GDM diagnosis using the IADPSG2010/WHO2013 and early β-cell function was associated with an even higher risk of pre-diabetes. While it is not yet clear whether early intervention during pregnancy can prevent the development of future type 2 diabetes in GDM women, such information could be useful in raising awareness of increased long-term risk. Indeed, future studies that examine if early intervention based on impaired β-cell function in the first trimester could prevent development of type 2 diabetes could be warranted.

Limitations to our study include a modest sample size and loss of participants in the follow-up study. The study was collected in a city and included only women with Scandinavian heritage and our findings may therefore not apply to all demographics and ethnicities. The diagnosis of the GDM was performed later than the standard protocol, at 30–32 weeks and not at 24–28 weeks as recommended, although the prevalence of GDM suggests that this is an average risk cohort when it comes to GDM risk. In Norway an early HbA1c (before 16 weeks gestation) is suggested to be analyzed in women with risk factors, since this marker has been shown promising at predicting GDM^27^. Unfortunately we do not have HbA1c data in this cohort and are not able to evaluate the performance of HbA1c. Finally, our findings do not apply to high risk populations and we cannot exclude that there is information in early OGTTs (e.g. shape information from glucose curves) that can be utilized to enhance early detection of GDM.

In conclusion, our data in a normal low-risk population, suggest that a 75 g OGTT at 14–16 weeks is not suitable to be used as a diagnostic or screening test to diagnose the women who are likely to develop GDM later or exclude women that are not likely to develop GDM later. However, it may give information about which women who will develop pre-diabetes and possibly type 2 diabetes. The uncertain clinical benefit and increased resources needed to perform an OGTT instead of a simple fasting glucose measurement, as well its inability to predict woman who will develop GDM, makes it questionable if performing routine OGTT early in normal low-risk pregnancy can be justified at this time.

## Electronic supplementary material

Supplementary File

## Data Availability

All data generated or analysed during this study are included in this published article (and its Supplementary Information file).
