# Supplementary material for: Prediction of Gestational Diabetes Mellitus and Pre-diabetes 5 Years Postpartum using 75 g Oral Glucose Tolerance Test at 14–16 Weeks’ Gestation
Source: Sci Rep. 2018 Sep 6;8:13392. doi: 10.1038/s41598-018-31614-z (PMC6127333; doi:10.1038/s41598-018-31614-z)

Supplementary file

**Prediction of Gestational Diabetes Mellitus and Pre-diabetes 5 Years Postpartum using 75g Oral Glucose Tolerance Test at 14-16 Weeks’ Gestation**

Tove Lekva, Kristin Godang, Annika E. Michelsen, Elisabeth Qvigstad, Kjersti Ringvoll Normann, Errol R. Norwitz, Pål Aukrust, Tore Henriksen, Jens Bollerslev, Marie Cecilie Paasche Roland, Thor Ueland

Supplemental Table 1. Common guidelines for the diagnosis of GDM at gestational week 24-28

| Organisation | Who is screened? | Method | Fasting plasma glucose (mmol/L) | Glucose challenge | 1-h plasma glucose (mmol/L) | 2-h plasma glucose (mmol/L) | 3-h plasma glucose (mmol/L) |
| --- | --- | --- | --- | --- | --- | --- | --- |
| WHO (1999) | Women with risk factors | One-step* | ≥ 7.0 | 75g OGTT | Not required | ≥ 7.8 |  |
| IADPSG (2010)$ | All women | One-step* | ≥ 5.1 | 75g OGTT | ≥ 10.0 | ≥ 8.5 |  |
| WHO (2013)# |  | One-step* | ≥ 5.1 | 75g OGTT | ≥ 10.0 | ≥ 8.5 |  |
| Norway (2017) | Women with risk factors | One-step* | ≥ 5.3 | 75g OGTT |  | ≥ 9.0 |  |
| ACOG | All women | Two-step** | ≥ 5.3 | 50g OGTT  100g OGTT | ≥ 7.8  ≥10.0 | ≥ 8.6 | ≥ 7.8 |
| ADA | High-risk women | One-step* | ≥ 5.1 | 75g OGTT | ≥ 10.0 | ≥ 8.5 |  |
|  | Non high-risk women | Two-step** | ≥ 5.3-5.8 | 50g OGTT  100g OGTT | ≥ 7.8  ≥10-10.6 | ≥ 8.6-9.2 | ≥ 7.8-8.0 |
| CDA | All women | Two-step (preferred)* | ≥ 5.3 | 50g OGTT  75g OGTT | ≥ 7.8  ≥10.6 | ≥9.0 |  |
|  |  | One step* | ≥ 5.1 | 75g OGTT | ≥10.0 | ≥8.5 |  |

*One value is sufficient for diagnosis. **Two or more values required for diagnosis. # At any time in pregnancy. $ Fasting glucose 5.1-6.9 mmol/L in early pregnancy is also used to diagnose GDM. WHO World Health Organization, IADPSG International Association of the Pregnancy Study Group, ACOG American Congress of Obstetricians and Gynecologists, ADA American Diabetes Associations, CDA Canadian Diabetes Association

Supplemental Table 2. Crosstab of the number of women diagnosed with GDM and non-GDM at week 14-16 and their diagnostic criteria.

|  | WHO | | IADPSG2010 | | WHO2013 | | Norway | |
| --- | --- | --- | --- | --- | --- | --- | --- | --- |
|  | GDM V3 | Non-GDM V3 | GDM V3 | Non-GDM V3 |  |  | GDM V3 | Non-GDM V3 |
| GDM V1 | 19  (True positive) | 1  (False positive) | 103  (True positive) | 15  (False positive) | 107  (True positive) | 12  (False positive) | 22  (True positive) | 24  (False positive) |
| Non-GDM V1 | 109  (False negative) | 817  (True negative) | 130  (False negative) | 698  (True negative) | 129  (False negative) | 698  (True negative) | 66  (False negative) | 832  (True negative) |

Supplemental Table 3. Crosstab of the number of women diagnosed with GDM at 14-16 weeks having a BMI>30 at week 14-16

|  | WHO | | IADPSG2010/WHO2013 | | Norway | |
| --- | --- | --- | --- | --- | --- | --- |
|  | BMI>30 | BMI<30 | BMI>30 | BMI<30 | BMI>30 | BMI<30 |
| GDM V1 | 6  Sens: 6.9 %  PPV: 30 % | 14 | 25  Sens: 28.7 %  PPV: 21 % | 94 | 12  Sens: 13.8 %  PPV: 26.1 % | 34 |
| Non-GDM V1 | 81 | 870  Spec: 98.4 %  NPV: 91.5 % | 62 | 790  Spec: 89.4 %  NPV: 92.7 % | 75 | 847  Spec: 96.1 %  NPV: 91.9 % |

Supplemental Table 4. Crosstab of the number of women diagnosed with GDM at 14-16 weeks with age>35 at week 14-16

|  | WHO | | IADPSG2010/WHO2013 | | Norway | |
| --- | --- | --- | --- | --- | --- | --- |
|  | Age>35 | Age<35 | Age>35 | Age<35 | Age>35 | Age<35 |
| GDM V1 | 6  Sens: 3.2 %  PPV: 30 % | 14 | 34  Sens: 18.1 %  PPV: 28.3 % | 86 | 12  Sens: 6.4 %  PPV: 26.1 % | 34 |
| Non-GDM V1 | 182 | 777  Spec: 98.2 %  NPV: 81.0 % | 154 | 705  Spec: 89.1 %  NPV: 82.1 % | 175 | 755  Spec: 95.7 %  NPV: 81.2 % |

Supplemental Table 5. Crosstab of the number of women diagnosed with GDM at 14-16 weeks having a LGA baby

|  | WHO | | IADPSG2010/WHO2013 | | Norway | |
| --- | --- | --- | --- | --- | --- | --- |
|  | LGA | AGA | LGA | AGA | LGA | AGA |
| GDM V1 | 7  Sens: 5.3 %  PPV: 35 % | 13 | 27  Sens: 20.1 %  PPV: 23.7 % | 87 | 8  Sens: 6.1 %  PPV: 18.6 % | 35 |
| Non-GDM V1 | 126 | 759  Spec: 98.3 %  NPV: 85.8 % | 107 | 684  Spec: 88.7 %  NPV: 86.5 % | 124 | 735  Spec: 95.5 %  NPV: 85.6 % |

Supplemental Table 6. Association between early β-cell dysfunction (as reflected by ISSI-2) GDM diagnosis according to different criteria and future (5 year follow-up) pre-diabetes.

|  | WHO1999 | | | | | IADPSG2010 | | | | | WHO2013 | | | | | NORWAY 2017 | | | | |
| --- | --- | --- | --- | --- | --- | --- | --- | --- | --- | --- | --- | --- | --- | --- | --- | --- | --- | --- | --- | --- |
| Step | Wald | Sig. | OR | 95% CI | | Wald | Sig. | OR | 95% CI | | Wald | Sig. | OR | 95% CI | | Wald | Sig. | OR | 95% CI | |
| ISSI-2 | 16.0 | <0.001 | 0.34 | 0.20 | 0.58 | 15.8 | <0.001 | 0.34 | 0.20 | 0.58 | 15.8 | <0.001 | 0.34 | 0.20 | 0.58 | 16.0 | <0.001 | 0.34 | 0.20 | 0.58 |
| ISSI-2 | 15.8 | <0.001 | 0.32 | 0.18 | 0.56 | 8.4 | 0.004 | 0.44 | 0.25 | 0.77 | 6.7 | 0.009 | 0.47 | 0.26 | 0.83 | 9.3 | 0.002 | 0.42 | 0.24 | 0.73 |
| GDM | 0.6 | 0.452 | 0.58 | 0.14 | 2.41 | 5.7 | 0.017 | 3.65 | 1.26 | 10.52 | 6.7 | 0.010 | 4.32 | 1.42 | 13.10 | 7.0 | 0.008 | 4.82 | 1.51 | 15.42 |
| ISSI-2 | 14.7 | <0.001 | 0.30 | 0.16 | 0.56 | 9.7 | 0.002 | 0.25 | 0.10 | 0.59 | 8.1 | 0.005 | 0.26 | 0.11 | 0.66 | 9.0 | 0.003 | 0.38 | 0.21 | 0.72 |
| GDM | 0.0 | 1.000 | 1.00 | 0.10 | 9.58 | 9.1 | 0.003 | 8.37 | 2.10 | 33.42 | 9.4 | 0.002 | 8.80 | 2.19 | 35.34 | 4.7 | 0.030 | 7.92 | 1.23 | 51.10 |
| ISSI-2*GDM | 0.3 | 0.579 | 1.53 | 0.34 | 6.97 | 3.3 | 0.070 | 2.87 | 0.92 | 8.95 | 2.7 | 0.099 | 2.65 | 0.83 | 8.43 | 0.4 | 0.521 | 1.59 | 0.39 | 6.55 |

**Supplementary Figure 1.** Test characteristics when combining different cut-offs of glucose from the 75g OGTT at 14-16 weeks and BMI (over 30) and age (over 35). Sensitivity, specificity, PPV and NPV across the different GDM diagnostic criteria using 1% (fasting) and 4% (60 and 120 minutes) decreases from standard 30-32 week OGTT cut-offs for (A) OGTT (B) OGTT+ BMI (C) OGTT +Age (D) OGTT + BMI or Age. The % decrease in glucose is shown in italic. Vertical text shows the actual glucose values at the different time-points.

**
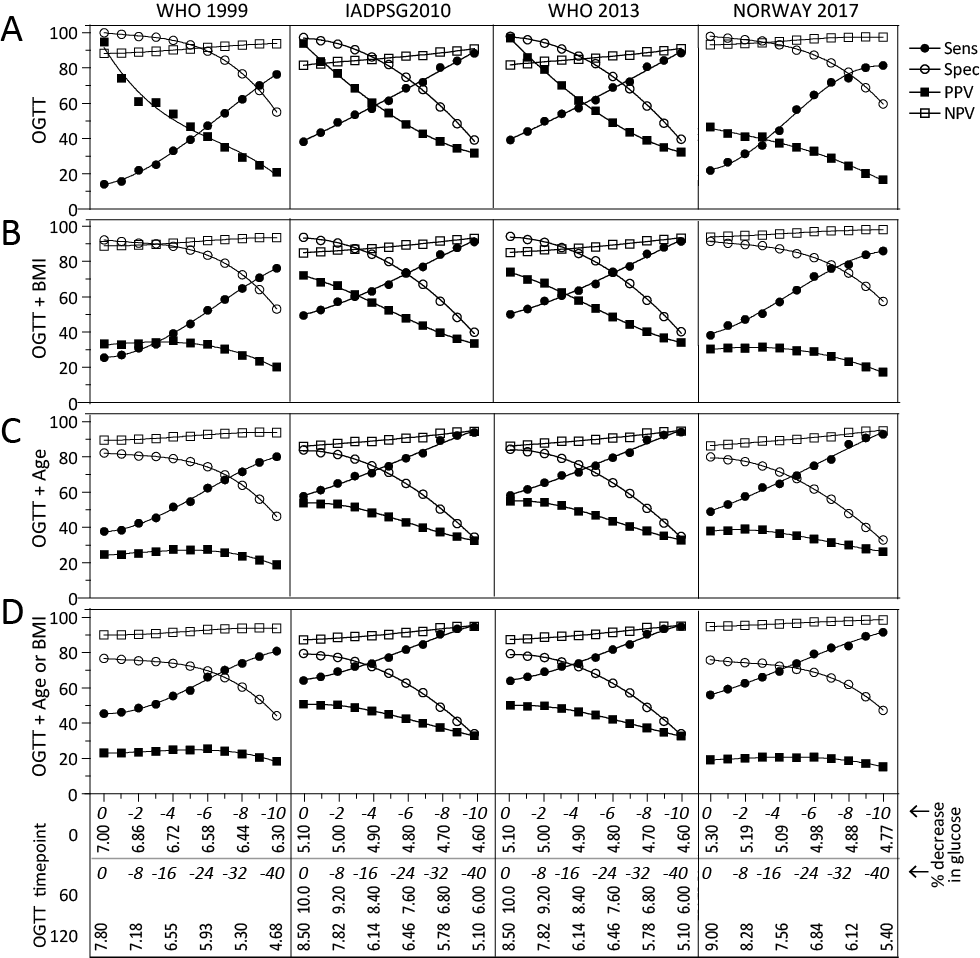
**

**Supplementary Figure 2. ROC curves of glucose and insulin measures at 14-16 weeks.**

Receiver operating characteristic (ROC) curves for predicting GDM using (A) WHO 1999, (B) IADPSG 2010, C) WHO 2013 and, D) Norway 2017 criteria by glucose and insulin measures at 14-16 weeks in pregnancy. *Sensitivity is expressed as 1-AUC for β-cell function, insulin sensitivity and insulinogenic index for comparison reasons. †weight gain from week 14-16 to 22-24.


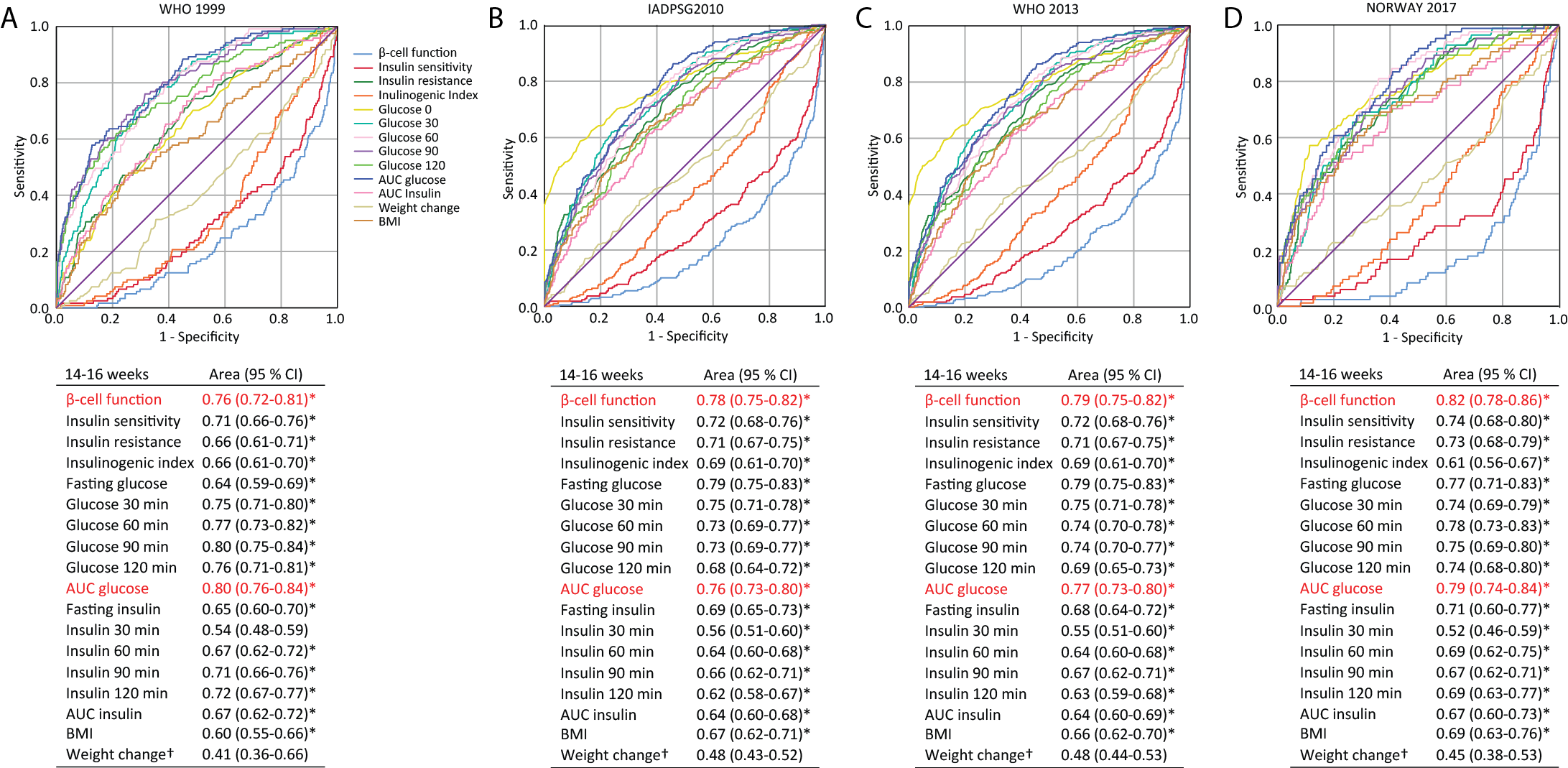

Supplement: Supplementary file 1 — Supplementary File [file 41598_2018_31614_MOESM1_ESM.docx]
